# Potential Effect Modifiers of the Association Between Physical Activity Patterns and Joint Symptoms in Middle‐Aged Women

**DOI:** 10.1002/acr.23430

**Published:** 2018-05-18

**Authors:** Geeske Peeters, Kimberley L. Edwards, Wendy J. Brown, Anna L. Barker, Nigel Arden, Anthony C. Redmond, Philip G. Conaghan, Flavia Cicuttini, Gita D. Mishra

**Affiliations:** ^1^ University of California, San Francisco, Trinity College Dublin, Dublin, Ireland, Monash University School of Public Health and Preventive Medicine, Melbourne, Victoria, Australia, University of Queensland School of Public Health, Brisbane, Queensland, Australia, and Arthritis Research UK Centre for Sport Exercise and Osteoarthritis Nottingham UK; ^2^ Arthritis Research UK Centre for Sport Exercise and Osteoarthritis and the University of Nottingham School of Medicine Nottingham UK; ^3^ University of Queensland School of Human Movement and Nutrition Sciences Brisbane Queensland Australia; ^4^ Monash University School of Public Health and Preventive Medicine Melbourne Victoria Australia; ^5^ Oxford University Arthritis Research UK Centre for Sport, Exercise, and Osteoarthritis Oxford UK; ^6^ Arthritis Research UK Centre for Sport Exercise and Osteoarthritis, Nottingham, UK, Leeds Institute of Rheumatic and Musculoskeletal Medicine, University of Leeds, and NIHR Leeds Biomedical Research Centre Leeds UK; ^7^ University of Queensland School of Public Health Brisbane Queensland Australia

## Abstract

**Objective:**

To examine whether body mass index (BMI), menopausal status, and hormone therapy (HT) use modify the association between physical activity (PA) patterns throughout middle age and the incidence and prevalence of joint symptoms in women in later middle age.

**Methods:**

Data were from 6,661 participants (born 1946–1951) in the Australian Longitudinal Study on Women's Health. Surveys, with questions on joint pain and stiffness, PA, height and weight, menopausal symptoms, and HT use, were completed every 3 years from 1998 to 2010. PA patterns were defined as none or low, low or meeting guidelines, fluctuating, or meeting guidelines at all times (reference pattern). Logistic regression was used to examine the association between PA patterns and prevalent (in 2010) and cumulative incident (1998–2010) joint symptoms and effect modification by patterns in BMI, menopausal status, and HT.

**Results:**

The groups representing fluctuating PA (odds ratio [OR] 1.34 [99% confidence interval (99% CI) 1.04–1.72]) and no or low PA (OR 1.60 [99% CI 1.08–2.35]) had higher odds of incident joint symptoms than those described as meeting guidelines at all times. Stratification by BMI showed that this association was statistically significant in the obese group only. No evidence for effect modification by menopausal status or HT use was found. The findings were similar for prevalent joint symptoms.

**Conclusion:**

Maintaining at least low levels of PA throughout middle age was associated with a lower prevalence and incidence of joint symptoms later in life. This apparent protective effect of PA on joint symptoms was stronger in obese women than in under‐ or normal‐weight women, and not related to menopause or HT status.

## Introduction

Middle age has been suggested to be a pivotal time for interventions to prevent chronic conditions, such as osteoarthritis (OA), whose incidence increases after the age of 50 years 1. Given its beneficial effects on cartilage health, physical activity (PA) is believed to be an important modifiable risk factor for OA 2. The complexity of the association between PA and OA is illustrated by findings suggesting that PA may have both protective and damaging effects on different joint structures 2. Despite decades of research, many aspects of the relationship between PA and OA‐related joint symptoms remain unclear 3. A better understanding of potential modifiers of the association between PA and joint symptoms may provide new insights into the etiology and guide the tailoring of preventive interventions.

**Significance & Innovations**

- Examination of physical activity patterns over 12 years in middle‐aged women showed that maintaining at least low levels of physical activity throughout middle age is required to benefit from its protective effects on joint symptoms in later middle age.
- The association between physical activity and joint symptom protection was modified by body mass index: the protective effect of physical activity on joint symptoms was stronger in obese women than in under‐ or normal‐weight women.
- This association was the same for subgroups according to menopause and hormone therapy use status.
- These findings support the use of strategies to improve physical activity interventions in middle‐aged women to prevent subsequent joint symptoms.

A study that followed middle‐aged women over a period of 12 years showed that being physically active at ages 52–58 years, but not at ages 47–52 years, reduced the risk of new‐onset joint symptoms in later middle age 4. The change in the association between PA and joint symptoms coincided with menopause 5. Premenopausal women may benefit from the protective effects of estrogen on joint structures and via the inflammation pathway 6. When estrogen levels decrease during the menopausal transition, inflammatory markers increase 7. The increase in inflammatory markers is partly explained by changes in fat mass 7. Low‐grade inflammation may affect joint structures via multiple pathways 8. There is some evidence that PA and body composition may influence the postmenopausal increase in inflammatory markers 7, and vice versa; menopause and body composition may modify the effects of PA on joint structures.

If estrogen has protective effects on joint structures, one would expect beneficial effects to arise from hormone therapy (HT) and detrimental effects following hysterectomy and oophorectomy. Hysterectomy and oophorectomy have been associated with an increased risk of OA 9, 10, 11. Evidence for an association between HT use and OA is inconclusive, however. Some 9, 12, but not all 12, 13, 14, observational studies found that HT use had significant protective associations with (radiographic) OA. In the studies that did not find statistically significant associations, the odds ratios were suggestive of protective associations of HT use, but the studies seemed underpowered to detect these 12, 13, 14. In contrast, other observational studies found that HT use was associated with increased risks of hip and hand, but not knee OA 15 or hip and knee replacement 16. Results from randomized controlled trials suggest that estrogen‐only therapy, but not estrogen plus progestin therapy, may be associated with reduced risks of joint replacement and joint symptoms 17, 18, 19.

In summary, there is a complex interplay between PA, body composition, and menopause and the risk of developing OA. Previous studies have typically examined PA at one point in time with outcomes measured at a later point in time. This approach does not account for the fluctuations in PA behavior over time 20. The aim of this prospective cohort study was to examine whether body composition, menopausal status, and use of HT modify the association between PA patterns throughout middle age and the incidence and prevalence of joint symptoms in later middle age in women.

## Materials and Methods

### Participants

Data were from the middle‐aged cohort (born 1946–1951) in the Australian Longitudinal Study on Women's Health (ALSWH), a prospective study of the health and well‐being of women 21. The sample was randomly drawn from the national Medicare health insurance database, which includes all Australian citizens and permanent residents, with intentional overrepresentation of women from rural and remote areas 21, 22. More details about the study can be found at http://www.alswh.org.au. The study was approved by the Ethics Committees of the Universities of Newcastle and Queensland. Informed consent was given by all participants.

**Figure 1:**
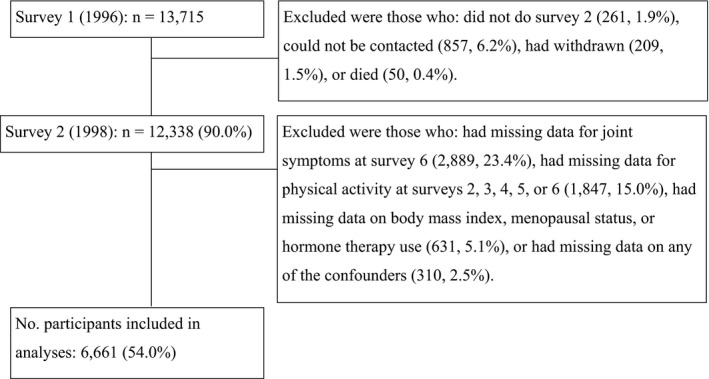
Flow chart of participants in the Australian Longitudinal Study on Women's Health included in the current analyses.

Baseline surveys were mailed in 1996, with the first followup in 1998 and then at intervals of 3 years to 2010. The baseline sample (n = 13,715, response rate 54%) was representative of Australian women in this age group 22. As the items for PA differed in the first survey, data from survey 2 (1998) to survey 6 (2010) were used for this article. The response rates for surveys 2 through 6 were 90.0%, 81.9%, 79.5%, 77.6%, and 73.0%, respectively. All participants with complete data on physical activity (1998–2010) and joint symptoms (2010) were included (Figure 1).

### Joint symptoms

In each survey, participants were asked to indicate the frequency of experiencing joint pain and stiffness in the last 12 months, with the response options being never, rarely, sometimes, and often. Having joint symptoms was defined as having joint pain and stiffness often at each survey 23. Two outcome variables were created. Prevalent joint symptoms was defined as reporting joint symptoms in 2010. For the cumulative incident definition, participants who reported having joint symptoms often at the 1998 survey were excluded and those reporting joint symptoms often at the 2001, 2004, 2007, or 2010 surveys were defined as having cumulative incident joint symptoms.

### PA

PA level was assessed at surveys 2–6 (1998–2010) using a modified version of the Active Australia questionnaire, which has acceptable measurement properties (test–retest correlation = 0.64, correlation with accelerometry = 0.52) 24. Participants were asked to report the duration, in the last week, of walking (for recreation, exercise, or transportation), moderate‐intensity leisure‐time activities (e.g., social tennis, recreational swimming, dancing), and vigorous‐intensity leisure‐time activities (activities that make you breathe harder or puff and pant, e.g., aerobics, competitive sport). The time spent in each activity (minutes/week) was multiplied by a metabolic equivalent (MET) score to reflect the average intensity of the activities in that category: 3.33 for walking briskly and moderate leisure‐time activities, and 6.66 for vigorous leisure‐time activity 25, 26. To estimate PA, the MET minutes/week in each category of activities were summed. The scores were categorized as none (<50), low (50 to <500) and meeting PA guidelines (≥500 MET minutes/week) 27. Participants were classified into 1 of 4 PA patterns according to their reported levels of PA at each of the 5 surveys: none or low, low or meeting guidelines, fluctuating, and meeting guidelines at all times (reference pattern). For additional details on these classifications, see Supplementary Table 1, available on the *Arthritis Care & Research* web site at http://onlinelibrary.wiley.com/doi/10.1002/acr.23430/abstract.

### Effect modifiers

Patterns of BMI, menopausal status, and HT use were based on self‐reported data from surveys 2–6. At each survey, BMI was calculated using self‐reported weight and height, and categorized as underweight/normal weight if BMI was <25 kg/m^2^ on at least 3 surveys, as overweight if BMI was 25 to <30 kg/m^2^ on at least 3 surveys, and as obese if BMI was ≥30 kg/m^2^ on at least 3 surveys. In accordance with the 2001 Stages of Reproductive Ageing Workshop criteria, participants were classified according to menopausal status at each survey 28. These classifications plus age at menopause were used to define average (ages >49 to <53 years), late (age ≥53 years), or early (age ≤49 years) age at menopause, and as oophorectomy + hysterectomy if they had bilateral oophorectomy with or without hysterectomy or as hysterectomy only (for details, see Supplementary Appendix A, available on the *Arthritis Care & Research* web site at http://onlinelibrary.wiley.com/doi/10.1002/acr.23430/abstract). Participants were classified as prolonged HT users if they reported HT use on 2 or more surveys and as nonusers/short‐term HT users if they reported HT use on no surveys or 1 survey.

### Sociodemographic and health variables

Sociodemographic and health variables were based on self‐report and measured at survey 2 (except level of education, which was only asked about at survey 1) and categorized as shown in Table 1. The number of chronic conditions (range 0–6) was assessed with the question “In the past 3 years, have you been diagnosed with or treated for: diabetes mellitus, heart disease, stroke, asthma/bronchitis, osteoporosis, or cancer?” Depressive symptoms were assessed using the 10‐item Center for Epidemiologic Studies Depression Scale (range 0–30, with higher scores indicating more symptoms) 29, 30. Copies of the surveys can be obtained from http://www.alswh.org.au/for-researchers/surveys.

**Table 1:** Sample characteristics in 1998^a^

Characteristic	Physical activity pattern 1998–2010				*P*
	None/low	Fluctuating	Low/MG	MG	
No. (%)	504 (7.6)	2,285 (34.3)	2,472 (37.1)	1,400 (21.0)	
Age, mean ± SD years	49.4 ± 1.4	49.5 ± 1.4	49.5 ± 1.5	49.5 ± 1.5	0.54
Living in urban areas	33.7	31.8	36.8	38.7	< 0.001
Married/de facto relationship	83.7	83.7	85.4	84.9	0.40
Level of education					< 0.001
No formal education	18.5	17.6	10.8	9.2	
Less than high school	39.1	33.9	29.3	29.8	
High school	13.1	17.5	17.8	15.2	
Trade/certificate/diploma	18.3	19.4	21.6	24.8	
University	11.1	11.5	20.5	21.0	
Paid work	59.7	59.5	63.2	60.7	0.05
Current smoker	19.8	17.2	12.2	9.5	< 0.001
No. chronic conditions, % ≥1	34.9	31.4	28.8	26.1	< 0.001
Depressive symptoms, median (IQR)	6 (3–10)	5 (3–10)	4 (2–8)	4 (1–7)	< 0.001
Joint symptoms	27.8	24.0	15.5	12.7	< 0.001
Body mass index					< 0.001
Under/normal weight	34.0	41.4	52.2	59.8	
Overweight	32.8	32.6	32.2	28.7	
Obese	33.2	26.0	15.6	11.4	
Menopausal status					0.004
Premenopause	34.0	34.0	34.5	37.0	
Perimenopause	25.5	24.3	26.7	27.0	
Postmenopause	11.7	14.0	14.8	12.7	
Oophorectomy + hysterectomy^b^	10.7	8.6	7.1	6.4	
Hysterectomy only	18.1	19.7	16.9	16.8	
Hormone therapy	28.8	23.5	21.3	18.5	< 0.001
Physical activity, median (IQR)^c^	0 (0–200)	400 (0–999)	600 (300–1,032)	1,399 (899‐2,198)	< 0.001

a Values are percentages unless otherwise indicated. MG = meeting guidelines; IQR = interquartile range.

b Includes women who had oophorectomy only (n = 40, 5.4%).

c In metabolic equivalent minutes/week.

### Statistical analysis

All analyses were done using Stata, version 11.1. Descriptive statistics were used to summarize sample characteristics for women in each of the PA pattern groups. Approximately normally distributed continuous variables were presented as means and SDs, and group differences were tested using analysis of variance. Non‐normally distributed continuous variables were presented as medians and interquartile ranges, and group differences were tested using the Kruskal‐Wallis test. Categorical variables were presented as percentages, and group differences were tested using the chi‐square test.

The association between PA patterns (1998–2010) and joint symptoms (2010) was analyzed using logistic regression. The analyses were performed for both prevalent and incident joint symptoms. Potential interaction with BMI, menopausal status, and HT use was examined by including a product term of each with the PA patterns and comparing the models with and without the product terms in terms of model fit using the likelihood ratio test. Potential collinearity between menopausal status and HT use was checked but not confirmed (variance inflation factors <1.1; 43.1% of those with oophorectomy/hysterectomy used HT). Next, the model was fitted for BMI strata, menopausal status, and HT use. Potential confounders were selected based on previous studies and included age, marital status, level of education, employment status, smoking status, chronic conditions, and depressive symptoms 2, 31, 32, 33, 34, 35. The association was adjusted for confounders (measured in 1998) that changed the regression coefficient by more than 10%, which was the case for depressive symptoms and level of education. The main analyses included data from women with complete data. As 309 participants had missing values on at least 1 of the potential confounders, multiple imputation by chained equations was used to impute these missing values 36, 37. Sensitivity analyses were done to examine the effect of exclusion due to missing values. An alpha level of 0.01 was set to account for multiple testing, and the data were expressed as odds ratios (ORs) with 99% confidence intervals (99% CIs).

## Results

**Figure 2:**
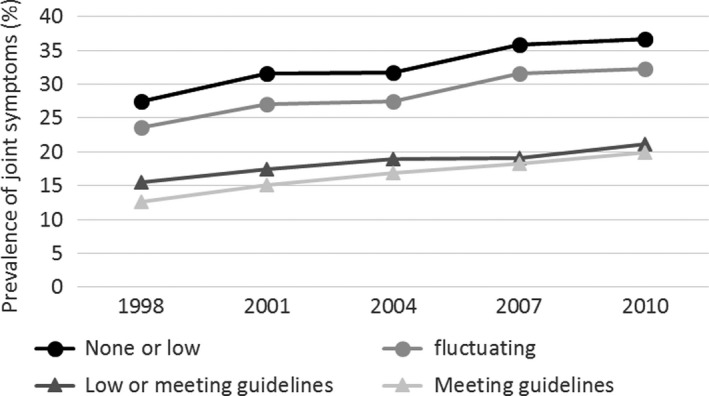
Prevalence of joint symptoms at each time point by pattern of physical activity over time (1998–2010). Figure based on data from women with complete data on physical activity and joint symptoms at all time points.

Based on 1998–2010 data, the 6,661 included participants were classified by their PA participation as follows: none or low (n = 504), fluctuating (n = 2,285), low or meeting guidelines (n = 2,472), and meeting guidelines at all times (n = 1,400) (Table 1). In 1998, the 4 groups did not differ in age (*P* = 0.54) and the participants had a mean ± SD age of 49.5 ± 1.5 years. Participants in the none or low and fluctuating groups were less likely to live in urban areas, less likely to have post–high school education, more likely to be current smokers, more likely to have chronic conditions, and had more depressive symptoms than participants in the low or meeting guidelines or meeting guidelines at all times groups (*P* ≤ 0.001). At study baseline, the none or low and fluctuating groups were also more likely to be obese, to have had oophorectomy and/or hysterectomy, and to use HT (*P* < 0.001). At the end of the followup in 2010, the prevalence of joint symptoms was markedly higher in the none or low (36.6%) and fluctuating (32.2%) groups than in the low or meeting guidelines (21.2%) and the meeting guidelines at all times (19.9%) groups (*P* < 0.001) (Figure 2).

After adjustment for BMI patterns, menopausal status, and HT use, and the confounders, the fluctuating group (OR 1.34 [99% CI 1.04–1.72]) and the none or low PA group (OR 1.60 [99% CI 1.08–2.35]) had higher odds of incident joint symptoms than those who were meeting guidelines at all times (Table 2). In the same model, overweight and obese participants had higher odds of reporting joint symptoms than underweight/normal‐weight participants. Also, participants using HT and those with hysterectomy only had higher odds of reporting joint symptoms than nonusers and participants with average age at menopause, respectively. Repeating these analyses for prevalent joint symptoms in 2010 yielded similar results for PA patterns, BMI, and HT use (Table 2). In addition, oophorectomy and/or hysterectomy were also associated with prevalent joint symptoms.

**Table 2:** Associations between patterns of physical activity, BMI, menopausal status, and HT use and joint symptoms in the total sample (n = 6,661)^a^

	Prevalent joint symptoms in 2010			Cumulative incident joint symptoms 1998–2010		
	No. (%)^b^	OR (99% CI)	*P*	No. (%)^b^	OR (99% CI)	*P*
Physical activity pattern						
MG at all times	1,400 (19.7)	1		873 (32.1)	1	
Low or MG	2,472 (21.2)	0.99 (0.79–1.23)	0.87	1,478 (32.6)	0.92 (0.72–1.17)	0.35
Fluctuating	2,285 (32.2)	1.43 (1.14–1.78)	< 0.001	1,246 (45.4)	1.34 (1.04–1.72)	0.003
None or low	504 (36.9)	1.61 (1.18–2.20)	< 0.001	262 (51.9)	1.60 (1.08–2.35)	0.002
BMI pattern						
Under/normal weight	2,759 (18.6)	1		1,665 (29.6)	1	
Overweight	2,240 (26.2)	1.48 (1.24–1.78)	< 0.001	1,344 (39.4)	1.49 (1.22–1.84)	< 0.001
Obese	1,662 (37.4)	2.12 (1.75–2.56)	< 0.001	850 (52.0)	2.26 (1.78–2.86)	< 0.001
Menopausal status pattern						
Average age at menopause	1,631 (20.1)	1		1,000 (32.5)	1	
Early age at menopause	1,103 (24.7)	1.19 (0.92–1.52)	0.08	635 (36.7)	1.08 (0.81–1.44)	0.48
Late age at menopause	1,807 (22.1)	1.15 (0.92–1.44)	0.11	1,122 (35.2)	1.11 (0.87–1.42)	0.28
Oophorectomy + hysterectomy	740 (36.5)	1.57 (1.19–2.08)	< 0.001	369 (49.1)	1.38 (0.97–1.97)	0.02
Hysterectomy only	1,380 (32.8)	1.53 (1.21–1.93)	< 0.001	733 (44.9)	1.36 (1.03–1.79)	0.004
HT pattern						
Nonuse/short‐term HT use	4,812 (23.1)	1		2,903 (34.8)	1	
Prolonged HT use	1,849 (33.1)	1.33 (1.12–1.58)	< 0.001	956 (47.5)	1.43 (1.15–1.78)	< 0.001

a BMI = body mass index; HT = hormone therapy; OR = odds ratio; 99% CI = 99% confidence interval; MG = meeting guidelines.

b Number of participants per category and percentage of participants reporting joint symptoms in 2010. In addition to the presented variables, the logistic regression model includes the confounders level of education, depressive symptoms, and chronic conditions.

Adding the product term of PA pattern and BMI significantly improved the model fit (likelihood ratio test *P* ≤ 0.01). Stratification by BMI showed that the association between PA pattern and joint symptoms was statistically significant in the obese group only for incident and prevalent joint symptoms (Table 3).

**Table 3:** Associations between physical activity patterns and joint symptoms fitted for stratification by body mass index^a^

PA pattern	Prevalent joint symptoms in 2010			Cumulative incident joint symptoms 1998–2010		
	No. (%)^b^	OR (99% CI)	*P*	No. (%)^b^	OR (99% CI)	*P*
Under/normal weight						
MG at all times	746 (15.6)	1		469 (27.5)	1	
Low or MG	1,111 (17.0)	0.99 (0.70–1.39)	0.95	702 (28.1)	0.93 (0.64–1.32)	0.55
Fluctuating	755 (22.8)	1.24 (0.86–1.77)	0.13	462 (33.6)	1.12 (0.76–1.66)	0.46
None or low	147 (23.8)	1.23 (0.68–2.20)	0.37	74 (35.1)	1.16 (0.57–2.35)	0.60
Overweight						
MG at all times	456 (22.4)	1		302 (36.4)	1	
Low or MG	856 (24.3)	1.07 (0.75–1.54)	0.63	506 (34.1)	0.85 (0.57–1.28)	0.31
Fluctuating	772 (28.4)	1.18 (0.82–1.70)	0.25	460 (45.0)	1.19 (0.79–1.80)	0.28
None or low	156 (37.2)	1.70 (1.00–2.91)	0.01	93 (48.4)	1.45 (0.76–2.76)	0.14
Obese						
MG at all times	198 (29.3)	1		115 (39.1)	1	
Low or MG	505 (25.0)	0.82 (0.50–1.34)	0.31	289 (41.5)	1.07 (0.59–1.93)	0.77
Fluctuating	758 (45.5)	1.96 (1.24–3.10)	< 0.001	350 (60.3)	2.21 (1.23–3.95)	< 0.001
None or low	201 (46.3)	1.92 (1.10–3.35)	0.003	97 (66.0)	2.74 (1.28–5.84)	0.001

a PA = physical activity; OR = odds ratio; 99% CI = 99% confidence interval; MG = meeting guidelines.

b Number of participants per category and percentage of participants reporting joint symptoms in 2010. The results are shown after adjustment for level of education, depressive symptoms, chronic conditions, menopausal status, and hormone therapy use.

Adding the product term of PA pattern and menopausal status did not significantly improve the model fit (likelihood ratio test *P* ≥ 0.27). Stratification by menopausal status showed that there were no statistically significant associations between PA pattern and joint symptoms within any of the strata (Table 4).

**Table 4:** Associations between physical activity patterns and joint symptoms fitted for stratification by menopausal status and HT use^a^

Physical activity pattern	Prevalent joint symptoms in 2010			Cumulative incident joint symptoms 1998–2010		
	No. (%)^b^	OR (99% CI)	*P*	No. (%)^b^	OR (99% CI)	*P*
Average age at menopause (49–53 years)						
MG at all times	376 (15.2)	1		258 (28.3)	1	
Low or MG	619 (16.0)	0.94 (0.58–1.51)	0.72	401 (27.9)	0.86 (0.54–1.38)	0.42
Fluctuating	521 (27.1)	1.57 (0.98–2.50)	0.01	278 (41.4)	1.47 (0.89–2.41)	0.05
None or low	115 (27.0)	1.45 (0.73–2.87)	0.16	63 (39.7)	1.31 (0.60–2.86)	0.37
Early age at menopause (≤49 years)						
MG at all times	206 (19.4)	1		129 (30.2)	1	
Low or MG	413 (19.1)	0.85 (0.48–1.51)	0.47	237 (33.3)	0.98 (0.52–1.87)	0.95
Fluctuating	400 (30.0)	1.25 (0.71–2.20)	0.30	229 (41.1)	1.18 (0.61–2.26)	0.52
None or low	84 (39.3)	1.78 (0.82–3.88)	0.06	40 (52.5)	1.60 (0.58–4.45)	0.24
Late age at menopause (≥53 years)						
MG at all times	404 (16.3)	1		259 (33.6)	1	
Low or MG	709 (19.5)	1.18 (0.76–1.83)	0.32	442 (29.9)	0.79 (0.51–1.24)	0.18
Fluctuating	570 (26.7)	1.41 (0.90–2.21)	0.05	344 (39.5)	1.01 (0.63–1.62)	0.94
None or low	124 (34.7)	1.79 (0.95–3.36)	0.02	77 (52.0)	1.43 (0.70–2.92)	0.20
Oophorectomy + hysterectomy						
MG at all times	123 (33.3)	1		74 (37.8)	1	
Low or MG	250 (28.0)	0.72 (0.39–1.34)	0.18	131 (40.5)	1.04 (0.47–2.34)	0.89
Fluctuating	283 (43.1)	1.26 (0.70–2.27)	0.32	127 (62.2)	2.15 (0.94–4.92)	0.02
None or low	75 (45.3)	1.13 (0.50–2.56)	0.69	37 (56.8)	1.64 (0.53–5.09)	0.26
Hysterectomy only						
MG at all times	282 (24.5)	1		153 (34.6)	1	
Low or MG	481 (28.5)	1.08 (0.68–1.70)	0.67	257 (39.7)	1.07 (0.61–1.88)	0.75
Fluctuating	511 (39.3)	1.49 (0.95–2.34)	0.02	268 (52.6)	1.54 (0.87–2.72)	0.05
None or low	106 (42.5)	1.66 (0.87–3.16)	0.05	45 (64.4)	2.56 (0.99–6.62)	0.01
Nonuse/short‐term HT use						
MG at all times	1,046 (16.9)	1		685 (31.0)	1	
Low or MG	1,795 (19.5)	1.09 (0.83–1.43)	0.42	1,109 (29.0)	0.83 (0.63–1.10)	0.09
Fluctuating	1,630 (29.1)	1.48 (1.13–1.94)	< 0.001	927 (42.3)	1.27 (0.95–1.69)	0.03
None or low	341 (31.4)	1.55 (1.06–2.29)	0.001	182 (45.6)	1.34 (0.84–2.12)	0.10
Prolonged HT use						
MG at all times	354 (28.0)	1		188 (36.2)	1	
Low or MG	677 (25.6)	0.80 (0.54–1.19)	0.15	369 (43.4)	1.22 (0.75–1.99)	0.30
Fluctuating	655 (39.9)	1.31 (0.90–1.95)	0.07	319 (54.2)	1.61 (0.97–2.68)	0.02
None or low	163 (48.5)	1.70 (1.00–2.89)	0.01	80 (66.3)	2.60 (1.23–5.50)	0.001

a HT = hormone therapy; OR = odds ratio; 99% CI = 99% confidence interval; MG = meeting guidelines.

b Number of participants per category and the percentage of participants reporting joint symptoms in 2010. Results shown are after adjustment for level of education, depressive symptoms, chronic conditions, and body mass index. The models fitted for stratification by HT use were additionally adjusted for menopausal status.

Adding the product term of PA pattern and HT use did not significantly improve the model fit (likelihood ratio test *P* ≥ 0.17). Stratification by HT use showed that the association between PA pattern and joint symptoms was statistically significant in the HT prolonged users group for incident joint symptoms and in nonusers/short‐term users group for prevalent joint symptoms (Table 4).

Participants with complete data were more likely to have higher levels of education than participants with missing data on confounders (*P* = 0.001), but there were no statistically significant differences in other sociodemographic and health characteristics. After imputation of the missing values on confounders, no appreciable difference in the ORs compared with the complete case analysis was found (see Supplementary Tables 2–4, available on the *Arthritis Care & Research* web site at http://onlinelibrary.wiley.com/doi/10.1002/acr.23430/abstract). Compared with participants with missing data on any of the variables, those with complete data were more likely to live in urban areas, be married, have higher levels of education, be employed, be nonsmokers, and have a late menopause (*P* < 0.001). Participants with complete data also had fewer depressive symptoms and chronic conditions (*P* < 0.001), but there were no significant differences in the prevalence of joint symptoms, BMI patterns, or HT use.

## Discussion

Examination of PA patterns over 12 years in middle‐aged women showed that consistently throughout middle age at least low levels of PA are required to benefit from its protective effects on joint symptoms. The protective effect of PA on joint symptoms seemed stronger in obese women than in underweight/normal weight or overweight women, and this effect appears unrelated to menopause status and HT use.

To our knowledge, no other studies have examined the association between patterns of PA over time and joint symptoms in this age group. The finding that being physically active is associated with lower odds of joint symptoms is consistent with previous findings from the same cohort 4, 38. As there were no significant differences between the low or meeting guidelines and the meeting guidelines at all times groups (Table 2), it seems that doing at least low levels of PA consistently at each time point is sufficient for protective effects on joint symptoms. The current results further suggest that the low levels of PA need to be sustained over time to be beneficial.

Although the relationship between high BMI and increased risk of joint symptoms is well established 39, 40, our findings shed new light on the potential modifying role of BMI on the association between PA and joint symptoms in middle‐aged women. Contrary to previous studies, which found no evidence of effect modification by BMI in the association between PA and OA 33, 35, 41, we found stronger associations between PA and joint symptoms in the obese group than in the underweight/normal‐weight group. Previous studies may have been underpowered, however, to detect significant associations within each of the strata, because stratification led to small numbers of events in each category. This finding is particularly of importance, as it counters the concern that PA may increase the risk of joint symptoms in obese people due to joint loading.

The relationship between menopausal status and joint symptoms is less clear. In line with other studies, our findings suggest that early or late natural progression through menopause does not seem to be associated with the risk of joint symptoms, OA, or OA‐related joint replacement surgery 16, 42, 43. In contrast, in an Italian study of 42,464 women who consulted the clinic for menopausal problems, those who were postmenopausal were more likely to report having been diagnosed with and treated for OA (OR 1.18 [95% CI 1.08–1.28) 9. It may be that the negative current and previous findings were underpowered to detect a small effect of natural menopause on joint health. Alternatively, as the Italian study involved cross‐sectional analyses of data from a selective sample of women experiencing menopausal problems, their results may not be generalizable to the general female population.

Women with hysterectomy and oophorectomy had higher risks of having (i.e., prevalence) and developing (i.e., incidence) joint symptoms than women with natural menopause. These findings are in line with those from a case–control study 11 and possibly lend support to the hypothesis that estrogen deprivation increases the risk of developing OA. If this hypothesis is correct, then one would also expect a protective effect from HT use and from late menopause on joint symptoms. In contrast, the current findings suggest a negative association between HT use and joint symptoms and no association between age at menopause and joint symptoms. These findings are in line with those of another study of postmenopausal women in which HT users had higher odds of clinical OA of the hip, hand, and knee than nonusers 15. An explanation for these findings may be that HT use is a marker for other health problems, such as a decline in physical functioning 44, that coincide with joint symptoms. However, other studies have found protective effects of HT use on knee articular cartilage 45, radiographic OA 12, and joint replacements 16. These contrasting findings, from studies with different measures of joint health, make it difficult to draw any conclusions. Further research examining the effects of duration and type of HT is needed to better understand the association between HT use and joint health and the underlying mechanisms. The current results suggest that the association between physical activity and joint symptoms is modified by BMI, but not by menopausal status or HT use. Given that the prevalence of joint symptoms was highest (28.2%) and the proportion of women meeting PA guidelines was lowest (12.5%) in obese women, promoting PA in this group appears to be particularly important.

Strengths of this study include the large sample size and repeated measurements, which allowed for examination of PA patterns, BMI, menopausal status, HT use, and joint symptoms over time. However, due to the high prevalence of joint symptoms at the start of the followup (survey 2) there were relatively few cases with incident joint symptoms. This resulted in limited power to detect statistically significant associations with incident joint symptoms, particularly in the stratified analyses. A limitation of the study is that all measures, including the outcome, are based on self‐report. The outcome is a crude measure of frequency of any joint pain and stiffness perceived in the past 12 months. Information about which joints were affected or severity is not available. The strength of the association between PA and joint symptoms may vary by type of symptom (e.g., pain, swelling, stiffness), by joint, or by severity. The outcome may have included non‐arthritis‐related joint symptoms. We chose to examine joint symptoms rather than using a formal OA diagnosis, as many women in this age range may not have been diagnosed formally, using, for example, medical imaging. Previous research in this sample showed that fluctuations in joint symptoms coincided with within‐person variation in the self‐report of OA 23. Two definitions of joint symptoms were used: prevalent joint symptoms (reflecting the presence of symptoms at the end of the followup, irrespective of symptoms perceived during followup), and cumulative incident joint symptoms (reflecting the presence of joint symptoms during followup after the exclusion of those reporting symptoms at the start of the followup). With either definition, reverse causation cannot be fully ruled out, as participants may have developed joint symptoms during followup and subsequently altered their PA. We considered using a “pure” incident measure of joint symptoms, reflecting newly reported symptoms at the end of followup (survey 6) and excluding participants who reported joint symptoms during followup (surveys 2–5), so that the outcome followed the exposure. However, this resulted in the exclusion of a large proportion of the inactive participants, as they were more likely to develop symptoms at earlier ages. This then led to skewed results due to a healthy survivor bias. Self‐report of PA may have led to misclassification due to under‐ or overreporting. However, the survey used has been found to have acceptable validity when compared with accelerometry (correlation = 0.52) 24. In addition, the definitions of PA patterns used were driven by statistical criteria more than clinically relevant criteria. Various definitions of patterns were explored, but resulted in too many categories with small numbers of participants, as at each survey 37–45% of participants changed from the previous survey in the level of PA they reported (with 76% of participants changing at any survey interval). For example, consideration was given to splitting up the fluctuating group into separate “increasing” and “decreasing” patterns. However, variation within those categories regarding the timing and amount of increase and decrease, and the small numbers of participants in each of the categories, limited meaningful analyses of this classification. The effect modifiers BMI, menopausal status, and HT use were measured in 1998 only, and people may have changed categories during followup. This may have reduced the contrast between categories. Conversely, creating additional categories reflecting transitions between categories over time would have resulted in more categories with small numbers of participants. The analyses were adjusted for important confounders; however, residual confounding cannot be ruled out. Finally, the original sample was representative of Australian women aged 45–50 years, but with a somewhat higher representation of partnered women and women with education beyond the high school level 22. Comparison of women included in the analyses and those excluded due to missing values on the confounders did not show any differences in sociodemographic and health variables. Moreover, sensitivity analyses after imputing missing values on confounders did not alter the interpretation of the findings. However, most participants were excluded due to missing values on joint symptoms and PA. Compared with participants with any missing data, including PA and joint symptoms, those included in the analyses were in better socioeconomic positions, were healthier, and had healthier lifestyles. Hence, the current findings represent a somewhat more affluent and healthier population.

In conclusion, the results suggest that at least low levels of PA should be maintained throughout middle age for women to benefit from the protective effects on joint symptoms in later middle age. Furthermore, the association between PA and joint symptoms is modified by BMI, but not by menopausal status or HT use. Particularly in obese women, a physically active lifestyle contributes to reducing the risk of developing joint symptoms.

## Author Contributions

All authors were involved in drafting the article or revising it critically for important intellectual content, and all authors approved the final version to be submitted for publication. Dr. Peeters had full access to all of the data in the study and takes responsibility for the integrity of the data and the accuracy of the data analysis.

### Study conception and design

Peeters, Edwards, Brown, Barker, Arden, Redmond, Conaghan, Cicuttini, Mishra.

### Acquisition of data

Peeters, Mishra.

### Analysis and interpretation of data

Peeters, Edwards, Brown, Barker, Arden, Redmond, Conaghan, Cicuttini, Mishra.

## Supporting information

Supplementary Appendix A

Supplementary Tables 1–4
