# Supplementary material for: Potential Effect Modifiers of the Association Between Physical Activity Patterns and Joint Symptoms in Middle‐Aged Women
Source: Arthritis Care Res (Hoboken). 2018 May 18;70(7):1012–21. doi: 10.1002/acr.23430 (PMC6033095; doi:10.1002/acr.23430)
Supplement: Supplementary file 1 — Supplementary Appendix A [file ACR-70-1012-s001.docx]

**Supplementary Appendix A.** *Definition of the menopausal status pattern across surveys 2 (1998) to 6 (2010).*

In accordance with the 2001 Stages of Reproductive Ageing Workshop criteria,[^28^](#_ENREF_28) participants were classified at each survey as:

-‘pre-menopausal’ if they menstruated in the last three months and no change in menstrual frequency in the last 12 months;

-‘peri-menopausal’ if they reported changes in menstrual frequency or 3-11 months of amenorrhea;

-‘post-menopausal’ if they reported amenorrhea for 12 consecutive months or more;

-‘bilateral oophorectomy + hysterectomy’ if they reported bilateral oophorectomy only or in combination with hysterectomy; and

-‘hysterectomy only’ if they reported hysterectomy without bilateral oophorectomy.

Participants were also asked the age at which the bleeding stopped permanently. This was taken as the age at menopause and longitudinally cleaned across surveys 2 to 6. To create the variable ‘menopausal status pattern’ the following set of rules was applied.

1. ‘Early age at menopause’ was defined as age at menopause of <=49 years;
2. ‘Average age at menopause’ was defined as age at menopause of 49-53 years;
3. ‘Late age at menopause’ was defined as age at menopause of >=53 years;
4. Participants who were pre- or peri-menopausal at surveys 2 to 5 (aged 56-61 years), had not had oophorectomy and/or hysterectomy and did not use HT or OCP, were classified as ‘late age of menopause’;
5. If ‘oophorectomy + hysterectomy’ was reported at survey 2, the participant was classified as ‘oophorectomy + hysterectomy’
6. If ‘hysterectomy only’ was reported at survey 2, the participant was classified as ‘hysterectomy only’
