# Supplementary material for: Potential Effect Modifiers of the Association Between Physical Activity Patterns and Joint Symptoms in Middle‐Aged Women
Source: Arthritis Care Res (Hoboken). 2018 May 18;70(7):1012–21. doi: 10.1002/acr.23430 (PMC6033095; doi:10.1002/acr.23430)
Supplement: Supplementary file 2 — Supplementary Tables 1–4 [file ACR-70-1012-s002.doc]

**Supplementary Table 1. Physical activity pattern classification**

| Pattern label | Definition | Examples | | | | |
| --- | --- | --- | --- | --- | --- | --- |
|  |  | ’98 | ’01 | ’04 | ’07 | ’10 |
| None or low | Classified as none or low levels of physical activity at all time points, with at least one survey classified as ‘none’. | none  none | none  low | none  low | none  low | none  low |
| Low or meeting guidelines | Low levels or meeting guidelines at all time points, with at least one survey classified as ‘low’. | low  low | low  MG | low  MG | low  MG | low  MG |
| Meeting guidelines at all times | Meeting guidelines for physical activity at all time points. | MG | MG | MG | MG | MG |
| Fluctuating | Fluctuating between levels of physical activity without fitting in any of the other patterns. | none | MG | low | none | low |

**Supplementary Table 2.** Associations between physical activity patterns, BMI, menopause, HT and joint symptoms in the total sample after imputation of missing values (n=6971)

|  | Prevalent joint symptoms in 2010 | | | | Cumulative incident joint symptoms from 1998 to 2010 | | | |
| --- | --- | --- | --- | --- | --- | --- | --- | --- |
|  | n (%)* | OR | 99%CI | p | n (%)* | OR | 99%CI | P |
| Physical activity pattern |  |  |  |  |  |  |  |  |
| MG at all times | 1458 (19.6) | 1 |  |  | 897 (32.1) | 1 |  |  |
| Low or MG | 2582 (21.1) | 0.99 | 0.79-1.23 | 0.87 | 1540 (32.9) | 0.93 | 0.73-1.18 | 0.43 |
| Fluctuating | 2399 (32.3) | 1.43 | 1.15-1.78 | <0.001 | 1303 (45.3) | 1.33 | 1.04-1.70 | 0.003 |
| None or low | 528 (37.3) | 1.65 | 1.22-2.23 | <0.001 | 273 (52.7) | 1.67 | 1.14-2.44 | 0.001 |
| BMI pattern |  |  |  |  |  |  |  |  |
| Under/normal weight | 2890 (18.5) | 1 |  |  | 1735 (29.7) | 1 |  |  |
| Overweight | 2337 (26.1) | 1.48 | 1.23-1.77 | <0.001 | 1395 (39.6) | 1.49 | 1.22-1.82 | <0.001 |
| Obese | 1744 (37.7) | 2.13 | 1.77-2.57 | <0.001 | 883 (52.2) | 2.24 | 1.78-2.82 | <0.001 |
| Menopausal status pattern |  |  |  |  |  |  |  |  |
| Average age at menopause | 1700 (20.3) | 1 |  |  | 1030 (32.7) | 1 |  |  |
| Early age at menopause | 1155 (24.3) | 1.14 | 0.90-1.46 | 0.16 | 664 (36.9) | 1.08 | 0.81-1.42 | 0.51 |
| Late age at menopause | 1896 (22.5) | 1.16 | 0.94-1.44 | 0.07 | 1162 (35.5) | 1.12 | 0.88-1.42 | 0.24 |
| Oophorectomy+hysterectomy | 780 (36.0) | 1.53 | 1.17-2.00 | <0.001 | 392 (49.0) | 1.38 | 0.98-1.94 | 0.02 |
| Hysterectomy only | 1440 (32.8) | 1.51 | 1.20-1.90 | <0.001 | 764 (44.8) | 1.34 | 1.02-1.76 | 0.005 |
| HT pattern |  |  |  |  |  |  |  |  |
| Non/short term HT use | 5023 (23.2) | 1 |  |  | 3006 (35.0) | 1 |  |  |
| Prolonged HT use | 1948 (32.9) | 1.31 | 1.11-1.55 | <0.001 | 1007 (47.4) | 1.41 | 1.14-1.75 | <0.001 |

OR odds ratio; CI confidence interval; MG meeting guidelines; BMI body mass index; HT hormone replacement therapy

* Presented are the number of participants per category and the percentage of participants reporting joint symptoms in 2010.

In addition to the presented variables, the logistic regression model includes the confounders level of education, depressive symptoms and chronic conditions.

**Supplementary Table 3.** Associations between physical activity patterns and joint symptoms fitted for stratification by BMI and after imputation of missing values (n=6971)

|  | Prevalent joint symptoms in 2010 | | | | Cumulative incident joint symptoms from 1998 to 2010 | | | |
| --- | --- | --- | --- | --- | --- | --- | --- | --- |
| Physical activity pattern | n (%) | OR | 99%CI | p | n (%) | OR | 99%CI | p |
| Under/normal weight |  |  |  |  |  |  |  |  |
| MG at all times | 782 (15.3) | 1 |  |  | 474 (27.0) | 1 |  |  |
| Low or MG | 1163 (17.0) | 1.01 | 0.72-1.41 | 0.92 | 721 (28.2) | 0.97 | 0.68-1.37 | 0.80 |
| Fluctuating | 790 (22.8) | 1.26 | 0.89-1.79 | 0.09 | 461 (33.5) | 1.14 | 0.78-1.68 | 0.38 |
| None or low | 155 (24.5) | 1.30 | 0.74-2.30 | 0.23 | 78 (37.2) | 1.29 | 0.65-2.55 | 0.34 |
| Overweight |  |  |  |  |  |  |  |  |
| MG at all times | 471 (22.5) | 1 |  |  | 305 (36.7) | 1 |  |  |
| Low or MG | 894 (24.7) | 1.05 | 0.74-1.50 | 0.73 | 525 (34.3) | 0.84 | 0.56-1.25 | 0.26 |
| Fluctuating | 811 (28.2) | 1.15 | 0.80-1.65 | 0.32 | 472 (45.3) | 1.18 | 0.79-1.77 | 0.30 |
| None or low | 161 (36.6) | 1.65 | 0.97-2.79 | 0.01 | 93 (49.5) | 1.38 | 0.73-2.62 | 0.19 |
| Obese |  |  |  |  |  |  |  |  |
| MG at all times | 206 (29.1) | 1 |  |  | 118 (40.7) | 1 |  |  |
| Low or MG | 528 (25.0) | 0.83 | 0.51-1.34 | 0.31 | 294 (41.8) | 1.05 | 0.59-1.89 | 0.82 |
| Fluctuating | 798 (45.9) | 2.02 | 1.29-3.16 | <0.001 | 369 (59.9) | 2.09 | 1.18-3.69 | 0.001 |
| None or low | 212 (47.1) | 2.02 | 1.17-3.48 | 0.001 | 102 (67.6) | 2.91 | 1.38-6.13 | <0.001 |

OR odds ratio; CI confidence interval; MG meeting guidelines; BMI body mass index;

* Presented are the number of participants per category and the percentage of participants reporting joint symptoms in 2010.

The results are presented after adjustment for level of education, depressive symptoms, chronic conditions, menopausal status and HT use.

**Supplementary Table 4.** Associations between physical activity patterns and joint symptoms fitted for stratification by menopausal status and HT use, and after imputation of missing values (n=6971)

|  | Prevalent joint symptoms in 2010 | | | | Cumulative incident joint symptoms from 1998 to 2010 | | | |
| --- | --- | --- | --- | --- | --- | --- | --- | --- |
| Physical activity pattern | n (%)* | OR | 99%CI | p | n (%)* | OR | 99%CI | p |
| Average age at menopause |  |  |  |  |  |  |  |  |
| MG at all times | 394 (15.2) | 1 |  |  | 265 (28.3) | 1 |  |  |
| Low or MG | 643 (15.7) | 0.90 | 0.57-1.44 | 0.58 | 412 (28.2) | 0.88 | 0.55-1.40 | 0.48 |
| Fluctuating | 542 (27.7) | 1.57 | 0.99-2.49 | 0.01 | 287 (41.1) | 1.44 | 0.88-2.35 | 0.06 |
| None or low | 121 (28.1) | 1.51 | 0.78-2.92 | 0.11 | 67 (41.8) | 1.44 | 0.68-3.06 | 0.22 |
| Early age at menopause |  |  |  |  |  |  |  |  |
| MG at all times | 214 (18.7) | 1 |  |  | 132 (30.3) | 1 |  |  |
| Low or MG | 429 (18.6) | 0.87 | 0.49-1.53 | 0.52 | 247 (33.2) | 1.00 | 0.53-1.88 | 0.99 |
| Fluctuating | 424 (29.7) | 1.30 | 0.75-2.28 | 0.22 | 243 (41.6) | 1.23 | 0.65-2.32 | 0.41 |
| None or low | 88 (39.8) | 1.92 | 0.90-4.12 | 0.03 | 42 (52.4) | 1.63 | 0.60-4.43 | 0.20 |
| Late age at menopause |  |  |  |  |  |  |  |  |
| MG at all times | 427 (16.3) | 1 |  |  | 268 (33.6) | 1 |  |  |
| Low or MG | 742 (20.1) | 1.23 | 0.80-1.88 | 0.21 | 458 (30.1) | 0.81 | 0.52-1.26 | 0.22 |
| Fluctuating | 598 (27.1) | 1.43 | 0.93-2.22 | 0.03 | 358 (39.9) | 1.04 | 0.66-1.65 | 0.81 |
| None or low | 129 (34.9) | 1.79 | 0.97-3.32 | 0.02 | 78 (52.6) | 1.47 | 0.72-2.98 | 0.17 |
| Oophorectomy + hysterectomy | |  |  |  |  |  |  |  |
| MG at all times | 137 (33.6) | 1 |  |  | 76 (39.5) | 1 |  |  |
| Low or MG | 267 (27.7) | 0.70 | 0.39-1.30 | 0.14 | 142 (40.1) | 0.95 | 0.43-2.07 | 0.86 |
| Fluctuating | 298 (42.3) | 1.20 | 0.67-2.14 | 0.42 | 137 (61.3) | 1.86 | 0.83-4.15 | 0.05 |
| None or low | 78 (44.9) | 1.10 | 0.50-2.45 | 0.75 | 37 (56.8) | 1.48 | 0.48-4.56 | 0.37 |
| Hysterectomy only |  |  |  |  |  |  |  |  |
| MG at all times | 287 (24.3) | 1 |  |  | 156 (34.0) | 1 |  |  |
| Low or MG | 504 (28.4) | 1.08 | 0.69-1.69 | 0.67 | 281 (40.2) | 1.13 | 0.65-1.98 | 0.56 |
| Fluctuating | 537 (39.2) | 1.50 | 0.96-2.33 | 0.02 | 278 (51.8) | 1.54 | 0.88-2.70 | 0.05 |
| None or low | 112 (42.9) | 1.72 | 0.91-3.24 | 0.03 | 49 (65.3) | 2.77 | 1.10-6.98 | 0.004 |
| HT non-users |  |  |  |  |  |  |  |  |
| MG at all times | 1084 (16.7) | 1 |  |  | 701 (30.8) | 1 |  |  |
| Low or MG | 1877 (19.6) | 1.11 | 0.85-1.44 | 0.33 | 1151 (29.4) | 0.85 | 0.65-1.13 | 0.15 |
| Fluctuating | 1703 (29.5) | 1.53 | 1.18-1.99 | <0.001 | 963 (42.4) | 1.28 | 0.96-1.70 | 0.03 |
| None or low | 359 (31.8) | 1.62 | 1.11-2.36 | 0.001 | 191 (46.6) | 1.42 | 0.91-2.23 | 0.04 |
| HT users |  |  |  |  |  |  |  |  |
| MG at all times | 375 (28.0) | 1 |  |  | 196 (36.7) | 1 |  |  |
| Low or MG | 708 (25.4) | 0.79 | 0.54-1.16 | 0.11 | 389 (43.2) | 1.18 | 0.73-1.91 | 0.36 |
| Fluctuating | 696 (39.1) | 1.27 | 0.87-1.85 | 0.11 | 340 (53.5) | 1.53 | 0.93-2.51 | 0.03 |
| None or low | 169 (49.1) | 1.71 | 1.02-2.89 | 0.008 | 82 (67.1) | 2.62 | 1.25-5.50 | 0.001 |

OR odds ratio; CI confidence interval; HT hormone replacement therapy

* Presented are the number of participants per category and the percentage of participants reporting joint symptoms in 2010.

The results are presented after adjustment for level of education, depressive symptoms, chronic conditions and body mass index. The models fitted for stratification by menopausal status were additionally adjusted for HT use. The models fitted for stratification by HT use were additionally adjusted for menopausal status.
